# Insuficiência Cardíaca e Sarcopenia: O que Está no Meio?

**DOI:** 10.36660/abc.20230689

**Published:** 2023-11-14

**Authors:** Humberto Villacorta

**Affiliations:** 1 Universidade Federal Fluminense Niterói RJ Brasil Universidade Federal Fluminense, Niterói, RJ – Brasil

**Keywords:** Insuficiência Cardíaca, Sarcopenia, Prognóstico

A insuficiência cardíaca (IC) é uma doença progressiva e letal se não tratada corretamente.^1^ Pacientes com IC grave frequentemente evoluem para um estado de caquexia e perda de força muscular denominado sarcopenia.^2 - 5^ Sarcopenia e caquexia não são exatamente a mesma coisa. De acordo com o Consenso Europeu, a caquexia é caracterizada como grave perda de peso corporal, gordura, músculo e aumento do catabolismo proteico.^6^ Por outro lado, o termo sarcopenia é definido como perda de músculo esquelético relacionada à idade e declínio da força muscular e/ou desempenho físico, não necessariamente associado à perda de peso.^6^

Embora amplamente sobrepostas e por vezes difíceis de reconhecer, estas duas condições são altamente prevalentes em pacientes com IC grave. Nos Estudos que Investigam Comorbidades Agravantes da Insuficiência Cardíaca (SICA-HF), que analisaram 200 pacientes com fração de ejeção reduzida (69% dos casos) ou preservada, 32% apresentavam perda muscular. Destes, 30 (14,4%) apresentavam apenas sarcopenia, 25 (12%) apenas caquexia e 14 (6,7%) sarcopenia e caquexia juntas.^7^

**Figura 1:**
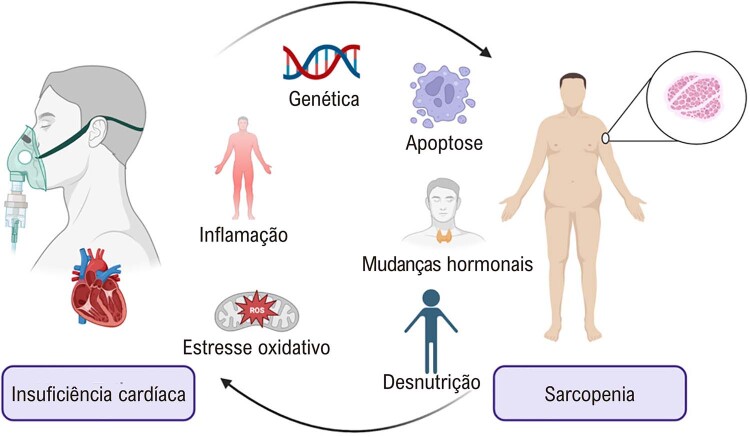
– A insuficiência cardíaca e a sarcopenia compartilham alguns fatores de risco. Pacientes com insuficiência cardíaca podem evoluir com sarcopenia pelos mecanismos apontados acima, e a sarcopenia indica pior prognóstico em pacientes com insuficiência cardíaca.

A fisiopatologia da relação entre IC e sarcopenia não é clara, mas partilham vias patogênicas semelhantes, como demonstrado na Figura 1 . Portanto, poderiam beneficiar-se de uma abordagem terapêutica comum. Uma vez que o paciente desenvolve sarcopenia, isso pode contribuir para o mau prognóstico da IC.^3^

Nesta edição dos Arquivos Brasileiros de Cardiologia, os autores investigaram genes centrais comuns em ambas as condições e previram vias associadas a esses genes por meio de análise quantitativa de bioinformática. O gene hub é um gene que está conectado a muitos outros genes em uma rede genética. Os autores usaram o Gene Expression Omnibus (GEO), um repositório público de dados genômicos funcionais. Eles descobriram que 114 genes expressos diferencialmente são comuns em ambas as condições. As vias relacionadas ao fator de crescimento, secreção de insulina e cGMP-PKG também foram enriquecidas na IC e na sarcopenia.^8^

Parabenizo os autores por seu esforço em iluminar uma área não totalmente compreendida. Apesar de algumas limitações já apontadas pelos autores, como o pequeno tamanho da amostra e o caráter retrospectivo do estudo, esses dados indicam alguns caminhos que podem ser utilizados para refinar o prognóstico na IC e servir como possível alvo terapêutico.
