# Supplementary material for: Insuficiência Cardíaca e Sarcopenia: O que Está no Meio?
Source: Arq Bras Cardiol. 2023 Nov 14;120(10):e20230689. [Article in Portuguese] doi: 10.36660/abc.20230689 (PMC10697681; doi:10.36660/abc.20230689)
Supplement: Translation

# Translation: Heart Failure and Sarcopenia: What is in between?

**Authors:** Humberto Villacorta

**Affiliations:** 1 Brazil Universidade Federal Fluminense, Niterói, RJ – Brazil

**Keywords:** Heart Failure, Sarcopenia, Prognosis

Heart failure (HF) is a progressive and lethal disease if not treated correctly.^1^Patients with severe HF often progress to a state of cachexia and loss of muscle strength called sarcopenia.^2 - 5^Sarcopenia and cachexia are not exactly the same thing. According to the European Consensus, cachexia is characterized as severe body weight, fat, muscle loss, and increased protein catabolism.^6^On the other hand, the term sarcopenia is defined as age-related loss of skeletal muscle and a decline in muscle strength and/or physical performance, not necessarily associated with weight loss.^6^

Although widely overlapping and sometimes difficult to recognize, these two conditions are highly prevalent in patients with severe HF. In the Studies Investigating Comorbidities Aggravating Heart Failure (SICA-HF), which analyzed 200 patients with either reduced (69% of the cases) or preserved ejection fraction, 32% had muscle wasting. Of these, 30 (14.4%) presented with sarcopenia alone, 25 (12%) with cachexia alone, and 14 (6.7%) with sarcopenia and cachexia together.^7^

The pathophysiology of the relationship between HF and sarcopenia is unclear, but they share similar pathogenic pathways, as demonstrated in Figure 1 . Therefore, they could benefit from a common therapeutic approach. Once the patient develops sarcopenia, it may contribute to the poor prognosis of HF.^3^

**Figure 1:** – Heart failure and sarcopenia share some risk factors. Patients with heart failure can evolve with sarcopenia through the mechanisms pointed out above, and sarcopenia indicates a worse prognosis in patients with heart failure.

In this Arquivos Brasileiros de Cardiologia issue, the authors investigated hub genes common to both conditions and predicted pathways associated with these genes through quantitative bioinformatics analysis. Hub gene is a gene that is connected to many other genes in a gene network. The authors used the Gene Expression Omnibus (GEO), a public repository of functional genomic data. They found that 114 differentially expressed genes are common to both conditions. Pathways related to growth factor, insulin secretion and cGMP-PKG were also enriched in HF and sarcopenia.^8^

I congratulate the authors for their effort to shed light on an area not fully understood. Despite some limitations already pointed out by the authors, such as the small sample size and the study’s retrospective nature, these data indicate some pathways that may be used to refine the prognosis in HF and serve as a possible therapeutic target.
